# Common targetable inflammatory pathways in brain transcriptome of autism spectrum disorders and Tourette syndrome

**DOI:** 10.3389/fnins.2022.999346

**Published:** 2022-12-15

**Authors:** Sarah Alshammery, Shrujna Patel, Hannah F. Jones, Velda X. Han, Brian S. Gloss, Wendy A. Gold, Russell C. Dale

**Affiliations:** ^1^Kids Neuroscience Centre, The Children’s Hospital at Westmead, Faculty of Medicine and Health, University of Sydney, Sydney, NSW, Australia; ^2^The Children’s Hospital at Westmead Clinical School, Faculty of Medicine and Health, University of Sydney, Sydney, NSW, Australia; ^3^Department of Neuroservices, Starship Children’s Hospital, Auckland, New Zealand; ^4^Khoo Teck Puat-National University Children’s Medical Institute, National University Health System, Singapore, Singapore; ^5^Westmead Research Hub, Westmead Institute for Medical Research, Westmead, NSW, Australia; ^6^The Brain and Mind Centre, The University of Sydney, Sydney, NSW, Australia

**Keywords:** inflammation, brain, bioinformatics, neurodevelopmental disorders, immune dysregulation

## Abstract

Neurodevelopmental disorders (NDDs), including autism-spectrum disorders (ASD) and Tourette syndrome (TS) are common brain conditions which often co-exist, and have no approved treatments targeting disease mechanisms. Accumulating literature implicates the immune system in NDDs, and transcriptomics of post-mortem brain tissue has revealed an inflammatory signal. We interrogated two RNA-sequencing datasets of ASD and TS and identified differentially expressed genes, to explore commonly enriched pathways through GO, KEGG, and Reactome. The DEGs [False Discovery Rate (*FDR*) <0.05] in the ASD dataset (*n* = 248) and the TS dataset (*n* = 156) enriched pathways involving inflammation, cytokines, signal transduction and cell signalling. Of the DEGs from the ASD and TS analyses, 23 were shared, all of which were up-regulated: interaction networks of the common protein-coding genes using STRING revealed 5 central up-regulated hub genes: *CCL2*, *ICAM1*, *HMOX1*, *MYC*, and *SOCS3*. Applying KEGG and Reactome analysis to the 23 common genes identified pathways involving the innate immune response such as interleukin and interferon signalling pathways. These findings bring new evidence of shared immune signalling in ASD and TS brain transcriptome, to support the overlapping symptoms that individuals with these complex disorders experience.

## Introduction

Neurodevelopmental disorders (NDDs), such as autism-spectrum disorders (ASD) and tic disorders including Tourette syndrome (TS), are neurological conditions which commonly co-exist and have shared genetic contributions (Clarke et al., 2012). ASD is characterised by social communication and language deficits, and repetitive stereotypical behaviour. Tics are repetitive stereotyped movements (motor tics) or vocalisations (vocal tics), and when present for more than 12 months, fulfil a diagnosis of TS. Tics are present in 11–22% of children with ASD, while ASD is present in 12% of children diagnosed with TS (Canitano and Vivanti, 2007; Pringsheim and Hammer, 2013; Darrow et al., 2017). Limited disease specific treatments are currently available for NDDs, and management focuses on symptom mitigation and developmental support (Wile and Pringsheim, 2013; Mittal, 2020).

The genetic aetiology of neurodevelopmental disorders is thought to be due to variants in multiple genes that converge on common pathways (Geschwind, 2008; Cross-Disorder Group of the Psychiatric Genomics Consortium, 2019). However, genetic aetiologies in these disorders are unable to explain the wide phenotypic heterogeneity, instead, the interaction between environmental and genetic factors are proposed to play an important role in pathogenesis of NDDs. In addition, immune dysregulation and inflammation have long been suggested to contribute to the pathophysiology, where early insults during gestation, such as maternal immune activation (MIA), can impact the development of the foetal brain (Scharf et al., 2013; Paschou et al., 2014; Sandin et al., 2014; Mataix-Cols et al., 2015; Frick et al., 2016; Tick et al., 2016; Autism Spectrum Disorders Working Group of The Psychiatric Genomics Consortium, 2017; Han et al., 2021). MIA, encompassing maternal conditions such as infection, asthma, obesity, autoimmune disease, and psychosocial stress, are associated with increased incidence of NDDs in offspring, such as ASD and TS (Dalsgaard et al., 2015; Jones et al., 2017, 2021; Patel et al., 2020). MIA is thought to act as a disease primer, which in addition to genetic predisposition, results in increased expression of neurodevelopmental disorders (Estes and McAllister, 2016). Studies have also shown dysregulation in proinflammatory cytokines such as IL-12, TNF, monocyte chemoattractant protein 2 (MCP-2), and IL-2 in the brains and peripheral blood of individuals with ASD and TS (Leckman et al., 2005; Vargas et al., 2005; Morer et al., 2010; Ashwood et al., 2011).

Transcriptomic analyses (RNA sequencing) of post-mortem brains from individuals with ASD have shown upregulated genes involved in inflammation and microglial dysregulation (Gandal et al., 2018a,b). Similarly, analysis of post-mortem brain striatum from individuals with TS identified up-regulated genes in immune and inflammatory pathways, and implicated microglial activation as a primary source of inflammation (Lennington et al., 2016). In both the ASD and TS brain transcriptome studies, the downregulated genes were enriched in pathways involved in synaptic function and GABA neurotransmission, aligning with the genetic variation found in these disorders (Lennington et al., 2016; Gandal et al., 2018a,b). By contrast, the upregulated inflammatory findings were considered more likely to be due to environmental factors or secondary (Lennington et al., 2016; Gandal et al., 2018a,b).

Given the shared genetic heterogeneity and comorbidity of NDDs, there is an increasing need to examine common disease pathways. As inflammation has been reported in brain transcriptomics in both ASD and TS, we examined for shared gene expression between ASD and TS in order to improve our understanding of the pathophysiology of NDDs and provide future potential therapeutic targets (Lennington et al., 2016; Gandal et al., 2018a,b).

## Materials and methods

### Data availability and open-source bioinformatic analysis

Human brain transcriptome data (RNA-seq) from two independent published studies were obtained with authors permission from synapse.org and analysed for differential gene expression and pathway enrichment analysis (Lennington et al., 2016; Gandal et al., 2018b). Unlike TS, where only one study interrogating the brain transcriptome exists, there are a number of studies investigating ASD brain transcriptome (Wright et al., 2017; Gandal et al., 2018a,b; Li et al., 2018; He et al., 2019). The current ASD dataset was chosen as it presented the largest cohort of samples (Gandal et al., 2018a,b). The ASD data were downloaded from synapse.org (ID: syn8234507) as count files, and RNA-seq metadata of 42 ASD cases were matched with 43 neurotypical controls (NC) (Gandal et al., 2018b). The pre-frontal cortex (PFC) region was chosen for the ASD analysis given the large sample size with matched controls. The TS data was downloaded as BAM files from synapse.org (ID: syn3158906), which included putamen and the caudate nucleus regions from 9 TS cases to 9 normal controls (Lennington et al., 2016). The bioinformatic workflow, including all utilised code and quality control figures can be found at https://github.com/sarahalshammery/ASDTS.

### Demographic and clinical variables of cases and controls

#### Autism spectrum disorder

A total of 42 ASD cases and 43 normal control PFC samples were utilised in this analysis (Supplementary Table 1; Gandal et al., 2018b). The ASD cohort selected (*n* = 42) consisted of nine female cases (21.43%) and 33 male cases (78.57%), with mean age of 26.38, median of 22.5, and range of 2–67 years. The normal control cohort selected (*n* = 43) comprised of nine females (20.93%) and 34 males (79.07%), with mean age of 28.63, median of 24, and range of 4–60 years. A Mann–Whitney test indicated no significant difference (*U* = 831, *P*-value = 0.5295) between the ages of the ASD and normal control cohorts. The full demographic data can be accessed from https://doi.org/10.7303/syn12080241.

#### Tourette syndrome

A total of 9 TS cases and 9 normal control caudate nucleus and putamen samples were included (Supplementary Table 1; Lennington et al., 2016). The TS cohort (*n* = 9) entailed four female cases (44.44%), and five male cases (55.56%) with mean age of 62.77, median of 52, and range of 29–84 years. The normal control (NC) cohort (*n* = 9) consisted of four (44.44%) females and five males (55.6%) with mean age of 58, median of 52, and range of 4–60 years. The full demographic data is in the Supplementary material of the original study [See their Supplementary Table 2 (Lennington et al., 2016)]. There was no statistical differences in the age of the TS cases in comparison to normal controls (Lennington et al., 2016).

### Data quality control

The ASD dataset was prepared and sequenced as described,^1^ reads were mapped against the Genome Reference Consortium Human Build 37 (GRCh37, otherwise known as hg19). The TS dataset were mapped against GRCh37 (hg19), and gene level counts for reference sequence (RefSeq) genes were assessed using HTSeq-count (Lennington et al., 2016). The raw counts for each dataset were converted to the counts per million (cpm) scale and filtered by expression using the *filterByExpr* function (Robinson et al., 2010). The data was normalised as per the EdgeR guide using Trimmed Mean of M-values (TMM) normalisation (Lennington et al., 2016).

### Differential gene expression analysis

Genes with an False Discovery Rate (*FDR*) of <0.05 following differential gene expression analysis of each dataset were considered differentially expressed genes (DEGs) in this investigation. The DEGs were identified by a quasi-likelihood (QL) negative binomial (NB) generalised log-linear model (glmQLF). Genes with a logFC > = 0 were considered to be up-regulated, and those below 0 were down-regulated. DEGs were visualised through a volcano plot using the ggplot 2 package (Wickham, 2016).

### Pathway and network enrichment analysis

Enrichments of the DEGs were identified through an over-representation analysis using Gene Ontology (GO) Biological Process, Reactome and the Kyoto Encyclopedia of Genes Genomes (KEGG), through the ClusterProfiler package [False Discovery Rate (*FDR*) <0.05] (Ashburner et al., 2000; Kanehisa and Goto, 2000; Yu et al., 2012; Kanehisa, 2019; The Gene Ontology Consortium, 2019; Jassal et al., 2020; Kanehisa et al., 2020). These are databases which allow genes to be grouped based on their relationships (GO), or the participation in pathways (Reactome and KEGG). For the main individual analyses, pathways enriched by less than 10 genes were excluded. Given the perceived more significant mechanistic insights of the Reactome results, they are presented in the main text, whereas GO and KEGG are presented in the Supplementary material.

The protein-coding DEGs which were common to both the ASD and the TS DGE analyses, were visualised using a protein-protein interaction (PPI) network through the Search Tool for the Retrieval of Interacting Genes/Proteins (STRING)^2^, with an interaction score >0.4, and default active interaction sources (Jassal et al., 2020). The PPI network from the DEGs common to both ASD and TS datasets were further imported into Cytoscape (Shannon et al., 2003). CytoHubba, an app for Cytoscape was used to identify hub genes by ranking nodes by network features through the multiple correlation clustering (MCC) method (Chin et al., 2014). The expression of the hub genes in the disease cohorts compared to controls were visualised using the ggplot 2 package (Wickham, 2016). A Shapiro–Wilk test was utilised to test normality of the hub genes’ counts.

## Results

### Transcriptional signatures

To identify relationships within the cases and their respective controls, we set out to explore differences based on transcriptome signatures. The ASD and TS cases were not observed to be transcriptionally distinct from their respective controls using hierarchal clustering analyses (Supplementary Figures 1, 2).

### Differential gene expression analysis

#### Autism spectrum disorder

**FIGURE 1:**
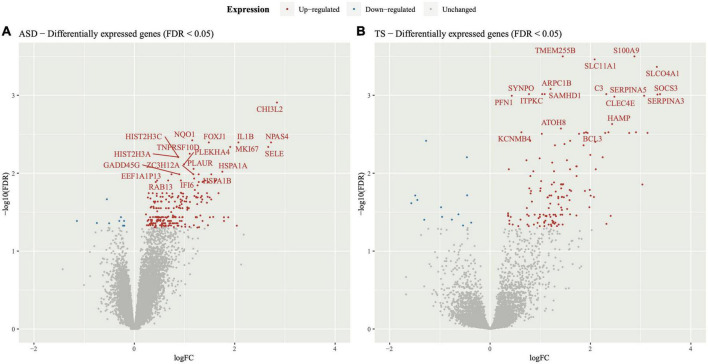
Volcano plot of differentially expressed genes in autism spectrum disorders (ASD) and Tourette syndrome (TS). Differential gene expression analysis was performed on the transcripts of **(A)** ASD cases and neurotypical controls and **(B)** TS cases and neurotypical control samples. The y-axis represents statistical significance (−log10FDR) and the x-axis represents gene expression log fold change (logFC). The top 20 differentially expressed genes were labelled for ease of viewing, all of which had an upregulated expression in both the ASD and TS datasets.

The DEGs within the PFC of ASD cases compared to neurotypical controls consisted of 239 upregulated genes and 9 downregulated genes, represented through a volcano plot (Figure 1A). Results of the DGE analysis can be accessed in Supplementary material (Supplementary Tables 2A,B).

#### Tourette syndrome

The DEGs within the striatum of individuals with TS compared to neurotypical controls consisted of 143 upregulated genes and 13 downregulated genes, as shown in the volcano plot (Figure 1B). Results of the DGE analysis can be accessed in Supplementary material (Supplementary Tables 3A,B).

### Immune pathways are enriched in autism spectrum disorders and Tourette syndrome brain transcriptome

#### Autism spectrum disorder

**FIGURE 2:**
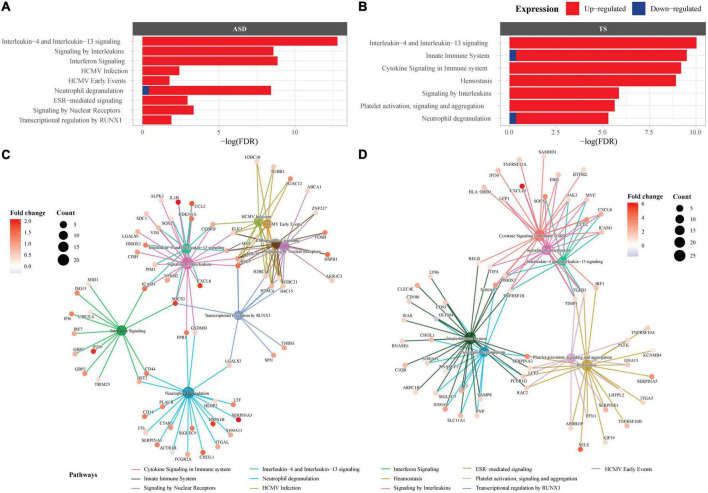
Pathway enrichment analysis in autism spectrum disorders (ASD) and Tourette syndrome (TS). Reactome enrichment analysis (*FDR* < 0.05) of the differentially expressed genes (DEGs) in the brain transcriptome of individuals with ASD compared with controls **(A,C)** and the DEGs of individuals with TS compared with controls **(B,D)**. Panels **(A,B)**: ASD enrichment results **(A)** and TS enrichment results **(B)** presented as bar plots of the top 10 pathways (y-axis), with the statistical significance of the pathways presented by the x-axis. Panels **(C,D)**: Connectivity network (CNET) plots of the top 5 enriched pathways and the interactions of genes that make up the pathways, represented by each gene’s fold change. The enriched pathways in ASD **(C)** and TS **(D)** are represented by a colour.

To explore enriched terms and pathways in the ASD DEGs, over-representation pathway analyses were conducted through three databases (*FDR* < 0.05). The GO analysis revealed 337 terms, consisting mainly of upregulated DEGs, and involved many immune response and inflammatory signalling, along with epigenetic terms (Supplementary Table 2C and Supplementary Figure 3). The top 3 GO terms were “humoral immune response,” “leukocyte mediated immunity,” and “lymphocyte mediated immunity.” Over-representation analysis using KEGG revealed 9 pathways, majority of which were enriched by up-regulated genes (Supplementary Figure 4). The top 3 KEGG pathways (based on *FDR*) were “Systemic lupus erythematosus,” “Neutrophil extracellular trap formation,” and “Staphylococcus aureus infection” (Supplementary Table 2D). Enrichment of the DEGs using Reactome revealed 9 pathways, mostly enriched by up-regulated DEGs (Figures 2A,C). Of the 9 pathways, the top 3 Reactome pathways (based on *FDR* and count) were “Interleukin-4 and Interleukin-13 signalling,” “Signalling by interleukins,” and “Interferon signalling.” Overall, 4/9 Reactome pathways were involved in the immune response consisting of cytokine signalling, innate and adaptive immune response pathways, 2/9 pathways were involved in signal transduction, 2/9 pathways were disease related, and 1/9 pathway belonged to gene expression and transcription. A full list of pathways from the three databases can be found in Supplementary material (Supplementary Tables 2C–E).

#### Tourette syndrome

The DEGs within the TS analysis enriched several terms and pathways from the three databases (*FDR* < 0.05). GO over-representation analysis revealed 135 terms, majority of which were enriched by up-regulated genes (Supplementary Table 3C and Supplementary Figure 5). The top 3 enriched GO terms were “immune response,” “cell activation,” and “leukocyte activation.” Over-representation analysis using KEGG did not enrich any pathways. Enrichment of the DEGs using Reactome revealed 7 pathways, most of which were enriched by up-regulated DEGs (Figures 2B,D). Of the 7 pathways, the top 3 Reactome pathways (sorted by *FDR* and count) were “Interleukin-4 and Interleukin-13 signalling,” “Innate Immune System,” “Cytokine Signalling in Immune system.” Overall, 5/7 Reactome pathways were involved in the immune response consisting of cytokine signalling, innate and adaptive immune response pathways, and 2/7 pathways were involved in the homeostasis pathway. The full list of pathways can be found in Supplementary material (Supplementary Tables 3C,D).

### Differentially expressed genes common to autism spectrum disorders and Tourette syndrome

**FIGURE 3:**
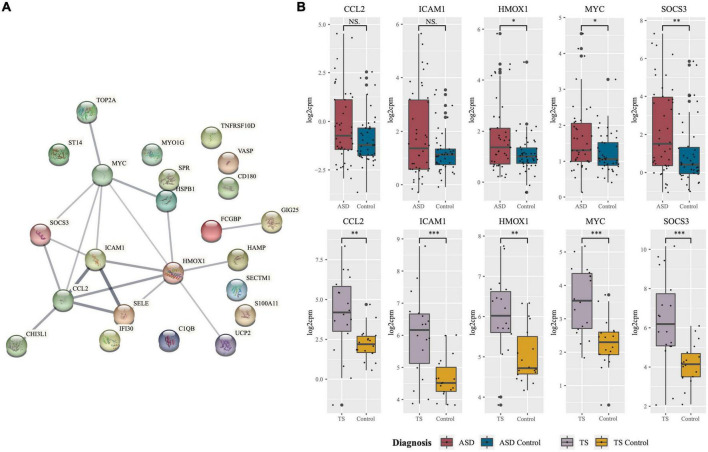
Protein network of common differentially expressed genes and expression of hub genes in autism spectrum disorders (ASD and Tourette syndrome (TS). **(A)** Protein-protein-interaction (PPI) network of genes found to be commonly differentially expressed in ASD and TS. The network consists of nodes (circles) and edges (lines) representative of predicted functional associations of the common protein-coding genes. Edge thickness is indicative of the strength of predicted evidence. **(B)** Hub genes central to the network were identified using Cytoscape, and the expression (log2cpm; y-axis) of the five hub genes in cases and neurotypical control (NC) cohorts. Expression of the five hub genes in ASD cases (*n* = 42; red) against neurotypical controls (*n* = 43; blue) and TS cases (*n* = 9; purple) and neurotypical controls (*n* = 9; yellow). The median is shown as the black line in each group’s box, with the small black dots representing each sample. The whiskers on either side of the boxes represent the minimum (Q1-1.5*IQR) and maximum (Q3 + 1.5*IQR) log2cpm excluding outliers. The big black dots represent potential outliers (***p < 0.001, **p < 0.01, *p < 0.05, NS, non-significant, Mann–Whitney–Wilcoxon Test). Network generated using STRING.

Of the DEGs from the ASD analysis, and the DEGs from the TS analysis, 23 DEGs were found to be shared. In both the ASD and TS datasets, 23/23 of the common genes had an up-regulated expression. The common protein-coding DEGs were mapped into a PPI network, and their expression in the ASD and TS cohorts was visualised (Figure 3A). From this network, we identified the top five hub genes using Cytoscape and CytoHubba, which consisted of C-C Motif Chemokine Ligand 2 (*CCL2*), Intercellular Adhesion Molecule 1 (*ICAM1*), Heme Oxygenase 1 (*HMOX1*), MYC Proto-Oncogene (*MYC*), and Suppressor Of Cytokine Signalling 3 (*SOCS3*; Table 1; Shannon et al., 2003; Chin et al., 2014). The raw data are presented in log scale for the five hub genes in cases compared to controls, shown for ASD and TS (Figure 3B). A full list of the common DEGs can be found in Supplementary material (Supplementary Table 4).

**TABLE 1:** Up-regulated hub genes in autism spectrum disorders (ASD) and Tourette syndrome (TS).

Gene	Gene name	Type of protein	Protein function	Reference
CCL2/MCP-1	C-C motif chemokine ligand 2/monocyte chemotactic and activating factor 1	Chemotactic cytokine.	Produced by microglia, neurons, astrocytes and mononuclear phagocytes, CCL2 recruits monocytes to the site of infection during inflammatory events.	Morer et al., 2010; Joly-Amado et al., 2020
ICAM1	Intercellular adhesion molecule 1	Immunoglobulin-like transmembrane glycoprotein expressed in the endothelial lumen.	Injury to the blood brain barrier results in microglia and astrocytes surrounding the capillary endothelial cells, where release of ICAM1 is responsible for eliminating antigens.	Müller, 2019
HMOX1	Heme oxygenase 1	Rate limiting enzymes that catalyses degradation of heme into biliverdin, ferrous ion, and carbon monoxide.	As a by-product of catabolising heme, HMOX1 has protective effects in vascular inflammation.	Araujo et al., 2012
MYC	Myelocytomatosis proto-oncogene	Transcription factor, binds DNA in a non-specific manner.	Involved in the regulation of immune checkpoints such as CD47 and PD-L1, and regulates expression of cells within the innate and adaptive immune responses.	Gnanaprakasam and Wang, 2017; Casey et al., 2018
SOCS3	Suppressor of cytokine Signalling 3	Suppressor of cytokine signalling family, part of a negative feedback system	Regulates cytokine signal transduction through STAT3 activation, using the gp130 receptor.	Carow and Rottenberg, 2014

Hub genes shared in ASD and TS following differential expression analyses. The common differentially expressed genes (23) from each disorder’s analysis were imported into STRING and Cytoscape to identify hub genes. The top 5 hub genes using the MCC method were selected.

### Common differentially expressed genes in autism spectrum disorders and Tourette syndrome enrich immune pathways

**FIGURE 4:**
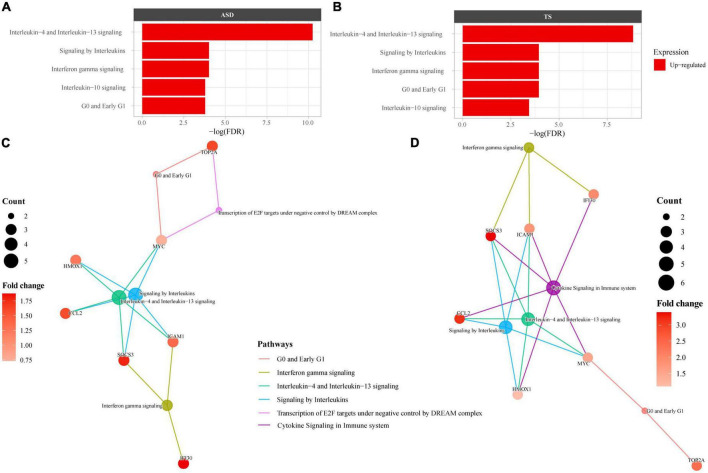
Overrepresented Reactome pathways common to autism spectrum disorders (ASD) and Tourette syndrome (TS). Reactome enrichment analysis (*FDR* < 0.05) of the common differentially expressed genes (DEGs) in the brain transcriptome of individuals with ASD compared with controls **(A,C)** and individuals with TS) compared with controls **(B,D)**. Panels **(A,B)**: ASD enrichment results **(A)** and TS enrichment results **(B)** presented as bar plots of the top 10 pathways (y-axis), with the statistical significance of the pathways presented by the x-axis. Panels **(C,D)**: Connectivity network (CNET) plots of the top 5 enriched pathways and the interactions of genes that make up the pathways, represented by each gene’s fold change. The enriched pathways in ASD **(C)** and TS **(D)** are represented by a colour.

As many of the enriched dysregulated pathways in ASD and TS overlapped, we set out to explore enriched pathways from the 23 DEGs common to both disorders, using overrepresentation analyses through Reactome. The Reactome analysis revealed up-regulated genes enriched in 6 pathways in ASD and 6 pathways in TS, with the top three common pathways involved in “Interleukin-4 and Interleukin-13 signalling,” “Interferon gamma signalling,” and “Signalling by Interleukins” (Figure 4). The full list of pathways can be found in the Supplementary material (Supplementary Table 4).

## Discussion

In this study we investigated enriched immune and inflammatory pathways in post-mortem brain tissue of individuals with ASD and TS, as well as pathways common to both disorders. As the focus of our hypothesis was to explore the immune response present in the seminal datasets, the paper’s focal point will be the inflammatory findings. Differential gene expression of the PFC region in ASD revealed that the majority (239 genes) of the 248 DEGs were upregulated compared to normal controls. Analogous to this, in the striatum of TS, the majority (143 genes) of the identified 156 DEGs were also upregulated compared to controls. This analysis validates the previous studies of upregulated genes in post-mortem brains of individuals with ASD and TS (Voineagu et al., 2011; Lennington et al., 2016).

The identified dominant signal of immune response and inflammation from the ASD GO enrichment analysis aligns with studies investigating brain transcriptome and pathology of individuals with ASD, and supports the involvement of astrocytes and activated microglia (Voineagu et al., 2011; Gandal et al., 2018b; Golovina et al., 2021). Of interest, the top 3 GO terms (by *FDR*) involved the humoral immune response and leukocyte mediated immunity. These terms were enriched by genes including *IL1b*, *TLR8*, complement genes (*C1QB*, *C1R*, *C2*), and chemokines (*CXCL5*, *CXCL8*)–all of which are involved in inflammation.

The enriched pathways established by the KEGG and Reactome analyses in the ASD cases identified major cellular pathways with therapeutic potential. The differential expression of central immune genes comprising cytokines, and CD cell markers (such as *IL1B*, *CD14*, *CD44*), support the reports of dysregulated cytokine levels in brains of individuals with ASD (Vargas et al., 2005; Li et al., 2009). Next, involvement of complement genes vital in phagocytosis (*C1QA*, *C1QB*, *C1QC*, *C1R*), which play a central role in immunity, response to infection, as well as synaptic pruning, further implicate the involvement of the immune system in ASD (Markiewski and Lambris, 2007; Dunkelberger and Song, 2010; Schafer et al., 2012). In addition, the enrichment of histone subunits fundamental to gene expression and epigenetic regulation (*H3C13*, *H3C7*, *H2BC11*, *H2BC3*), supports the concept of potential association between epigenetic regulation and inflammation (Weber-Stadlbauer, 2017).

Analysis of the TS differentially expressed genes using GO identified numerous enriched immune response and inflammatory signalling terms. The enriched pathways highlighted by the Reactome analysis in TS identified upregulated DEGs involved in the immune response such as cytokine signalling (*CXCL8*, *CXCL10*, *CCL2*) (Morer et al., 2010). In addition, pathways involving genes within major histocompatibility complexes II (i.e., *ICAM1*, *HLA-DRB1*) and the S100 family (*S100A9*, *S100A11*, *S100A12*) were enriched. These findings were similarly observed in the original analysis of these TS cases (Lennington et al., 2016).

Given the substantial comorbidity and overlap between NDDs, we identified genes and pathways common to both ASD and TS. We identified 23 common DEGs, all of which were upregulated in both disorders. From the 23 common genes, five were determined hub genes: *CCL2*, *ICAM1*, *HMOX1*, *MYC*, and *SOCS3*, all of which are involved in the immune response.

Our investigation has confirmed immune and inflammatory pathways are commonly enriched by up-regulated genes in ASD and TS. To further explore these intersecting findings, the 23 genes common to ASD and TS were analysed separately, which repeatedly identified enriched inflammatory pathways involving interleukin and interferon signalling. These pathways were enriched by the hub genes, which have a role in the immune response. We utilised this approach as it allowed for comparison of the same genes within both disorders, while employing the distinct *FDRs* from each analysis, offering insight into the strength of each disorder’s signal.

Our current study identified commonly enriched inflammatory pathways, however, several questions regarding the involvement of the immune response in ASD and TS remain unanswered. The cause of the identified inflammatory signals is still ambiguous, in addition to its nature. Research investigating the source of inflammation in NDDs has suggested it is an environmental or secondary component, rather than genetic (Voineagu et al., 2011; Lennington et al., 2016). In particular, the influence of MIA, which could create a neuroinflammatory environment in offspring, may alter immune signalling pathways and epigenetic control of cell function during the critical periods of development (Han et al., 2021). In addition, the identified inflammatory signal might be casual and pathogenic, or alternatively reactive or protective in origin, which cannot be deduced from the current investigation. Further functional and mechanistic explorations of tissue from individuals with NDDs might elucidate the nature of this inflammation.

Despite our findings, this study has a number of caveats. Firstly, our analysis involved different brain regions from the two disorders, prefrontal cortex for ASD, and caudate and putamen for TS, as corresponding brain region data was not available for the two disorders at the time of analysis.

Secondly, the majority of the samples within the two datasets were not children, as cohorts of paediatric post-mortem brain samples are scarce. Therefore, our analysis represents late-stage disease, and it is unclear if the findings will be reflected in younger cohorts. It is not known whether the inflammatory signal seen in ASD and TS accumulates over the course of life or is present in childhood.

Inflammation and the involvement of a dysregulated immune response is present in brain transcriptome data of both ASD and TS. Although classified as clinically distinct disorders, ASD and TS have common genetic aetiologies, along with overlaps in symptoms and comorbidities. We provide biological evidence that there is shared dysregulation of immune response and inflammatory signalling pathways in NDDs. Further studies to understand the cause and potential gene-environmental contribution to this inflammatory signal in these complex disorders is warranted.

## Data availability statement

Publicly available datasets were analysed in this study. This data can be found here: https://www.synapse.org/#!Synapse:syn12080241.

## Ethics statement

The studies involving human participants were reviewed and approved by National Institute of Mental Health. The patients/participants provided their written informed consent to participate in this study.

## Author contributions

SA analysed, interpreted, and wrote the results of this investigation. SP, HJ, VH, WG, and RD assisted in the interpretation and writing of the results. BG assisted in the analysis and interpretation of the results. All authors read and approved the final manuscript.

## Footnotes

1
http://www.doi.org/10.7303/syn4587615

2
https://string-db.org/

## Supplementary material

Supplementary Figure 1 Transcriptional clustering of ASD patients and normal controls. Cluster analyses identifying transcriptional differences within the autism-spectrum disorder (ASD; pink) patients and normal controls (NC; blue) using Euclidean distances. **(A)** Hierarchal cluster dendrogram using the agglomerative method. **(B)** Variance histogram identifies the amount of variance (y-axis) explained by each principal component (x-axis; dimension). **(C)** Principal component analysis (PCA) scatter plot of PC2 (y-axis) and PC1 (x-axis) which account for most of the variance in the data set. ASD, autism-spectrum disorder (*n* = 42), NC, normal control (*n* = 43).

Supplementary Figure 2 Transcriptional clustering of TS and normal controls. Cluster analyses identifying transcriptional differences within individuals with Tourette syndrome (TS; purple) and normal controls (NC; yellow) using Euclidean distances. **(A)** Hierarchal cluster dendrogram using the agglomerative method. **(B)** Variance histogram identifies the amount of variance (y-axis) explained by each principal component (x-axis; dimension). **(C)** Principal component analysis (PCA) scatter plot of PC2 (y-axis) and PC1 (x-axis) which account for most of the variance in the data set. TS, Tourette syndrome (*n* = 9), NC, normal control (*n* = 9).

Supplementary Figure 3 Autism spectrum disorders (ASD) Gene Ontology (GO) enrichment analysis. GO enrichment analysis (FDR/p.adjust < 0.05) of the top differentially expressed genes (*P* value < 0.05) in ASD. Statistical significance of the pathway (FDR) enriched is shown on the y-axis, while the enriched term is shown on the x-axis.

Supplementary Figure 4 Autism spectrum disorders (ASD) Kyoto Encyclopedia of Genes Genomes (KEGG) enrichment analysis. KEGG enrichment analysis (FDR/p.adjust < 0.05) of the top differentially expressed genes (*P* value < 0.05) in ASD. Statistical significance of the pathway (FDR) enriched is shown on the y-axis, while the enriched pathway is shown on the x-axis.

Supplementary Figure 5 Tourette syndrome Gene Ontology (GO) enrichment analysis. GO enrichment analysis (FDR/p.adjust < 0.05) of the top differentially expressed genes (*P* value < 0.05) in Tourette syndrome. Statistical significance of the pathway (FDR) enriched is shown on the y-axis, while the enriched term is shown on the x-axis.

## References

[B1] Araujo J. Zhang M. Yin F. (2012). Heme oxygenase-1, oxidation, inflammation, and atherosclerosis. *Front. Pharmacol.* 3:119. 10.3389/fphar.2012.00119 22833723 PMC3400084

[B2] Ashburner M. Ball C. Blake J. Botstein D. Butler H. Cherry J. (2000). Gene ontology: Tool for the unification of biology. The gene ontology consortium. *Nat. Genet.* 25 25–29. 10.1038/75556 10802651 PMC3037419

[B3] Ashwood P. Krakowiak P. Hertz-Picciotto I. Hansen R. Pessah I. Van de Water J. (2011). Elevated plasma cytokines in autism spectrum disorders provide evidence of immune dysfunction and are associated with impaired behavioral outcome. *Brain Behav. Immun.* 25 40–45. 10.1016/j.bbi.2010.08.003 20705131 PMC2991432

[B4] Autism Spectrum Disorders Working Group of The Psychiatric Genomics Consortium (2017). Meta-analysis of GWAS of over 16,000 individuals with autism spectrum disorder highlights a novel locus at 10q24.32 and a significant overlap with schizophrenia. *Mol. Autism* 8:21. 10.1186/s13229-017-0137-9 28540026 PMC5441062

[B5] Canitano R. Vivanti G. (2007). Tics and Tourette syndrome in autism spectrum disorders. *Autism* 11 19–28. 10.1177/1362361307070992 17175571

[B6] Carow B. Rottenberg M. (2014). SOCS3, a major regulator of infection and inflammation. *Front. Immunol.* 5:58. 10.3389/fimmu.2014.00058 24600449 PMC3928676

[B7] Casey S. Baylot V. Felsher D. (2018). The MYC oncogene is a global regulator of the immune response. *Blood* 131 2007–2015. 10.1182/blood-2017-11-742577 29514782 PMC5934797

[B8] Chin C. Chen S. Wu H. Ho C. Ko M. Lin C. (2014). cytoHubba: Identifying hub objects and sub-networks from complex interactome. *BMC Syst. Biol.* 8(Suppl. 4):S11. 10.1186/1752-0509-8-S4-S11 25521941 PMC4290687

[B9] Clarke R. Lee S. Eapen V. (2012). Pathogenetic model for Tourette syndrome delineates overlap with related neurodevelopmental disorders including autism. *Transl. Psychiatry* 2:e158. 10.1038/tp.2012.75 22948383 PMC3565204

[B10] Cross-Disorder Group of the Psychiatric Genomics Consortium (2019). Genomic relationships, novel loci, and pleiotropic mechanisms across eight psychiatric disorders. *Cell* 179 1469–1482.e11. 31835028 10.1016/j.cell.2019.11.020 PMC7077032

[B11] Dalsgaard S. Waltoft B. Leckman J. Mortensen P. (2015). Maternal history of autoimmune disease and later development of Tourette syndrome in offspring. *J. Am. Acad. Child Adolesc. Psychiatry* 54 495–501.e1. 10.1016/j.jaac.2015.03.008 26004665

[B12] Darrow S. Grados M. Sandor P. Hirschtritt M. Illmann C. Osiecki L. (2017). Autism spectrum symptoms in a Tourette’s disorder sample. *J. Am. Acad. Child Adolesc. Psychiatry* 56 610–617.e1. 10.1016/j.jaac.2017.05.002 28647013 PMC5648014

[B13] Dunkelberger J. Song W. (2010). Complement and its role in innate and adaptive immune responses. *Cell Res.* 20 34–50. 10.1038/cr.2009.139 20010915

[B14] Estes M. McAllister A. (2016). Maternal immune activation: Implications for neuropsychiatric disorders. *Science* 353 772–777. 10.1126/science.aag3194 27540164 PMC5650490

[B15] Frick L. Rapanelli M. Abbasi E. Ohtsu H. Pittenger C. (2016). Histamine regulation of microglia: Gene-environment interaction in the regulation of central nervous system inflammation. *Brain Behav. Immun.* 57 326–337. 10.1016/j.bbi.2016.07.002 27381299 PMC5012904

[B16] Gandal M. Haney J. Parikshak N. Leppa V. Ramaswami G. Hartl C. (2018a). Shared molecular neuropathology across major psychiatric disorders parallels polygenic overlap. *Science* 359 693–697. 29439242 10.1126/science.aad6469 PMC5898828

[B17] Gandal M. Zhang P. Hadjimichael E. Walker R. Chen C. Liu S. (2018b). Transcriptome-wide isoform-level dysregulation in ASD, schizophrenia, and bipolar disorder. *Science* 362:eaat8127. 30545856 10.1126/science.aat8127 PMC6443102

[B18] Geschwind D. (2008). Autism: Many genes, common pathways? *Cell* 135 391–395. 10.1016/j.cell.2008.10.016 18984147 PMC2756410

[B19] Gnanaprakasam J. Wang R. (2017). MYC in regulating immunity: Metabolism and beyond. *Genes (Basel)* 8:88. 10.3390/genes8030088 28245597 PMC5368692

[B20] Golovina E. Fadason T. Lints T. Walker C. Vickers M. O’Sullivan J. (2021). Understanding the impact of SNPs associated with autism spectrum disorder on biological pathways in the human fetal and adult cortex. *Sci. Rep.* 11:15867. 10.1038/s41598-021-95447-z 34354167 PMC8342620

[B21] Han V. Patel S. Jones H. Dale R. (2021). Maternal immune activation and neuroinflammation in human neurodevelopmental disorders. *Nat. Rev. Neurol.* 17 564–579. 10.1038/s41582-021-00530-8 34341569

[B22] He Y. Zhou Y. Ma W. Wang J. (2019). An integrated transcriptomic analysis of autism spectrum disorder. *Sci. Rep.* 9:11818. 10.1038/s41598-019-48160-x 31413321 PMC6694127

[B23] Jassal B. Matthews L. Viteri G. Gong C. Lorente P. Fabregat A. (2020). The reactome pathway knowledgebase. *Nucleic Acids Res.* 48 D498–D503. 10.1093/nar/gkz1031 31691815 PMC7145712

[B24] Joly-Amado A. Hunter J. Quadri Z. Zamudio F. Rocha-Rangel P. Chan D. (2020). CCL2 overexpression in the brain promotes glial activation and accelerates tau pathology in a mouse model of tauopathy. *Front. Immunol.* 11:997. 10.3389/fimmu.2020.00997 32508844 PMC7251073

[B25] Jones H. Han V. Patel S. Gloss B. Soler N. Ho A. (2021). Maternal autoimmunity and inflammation are associated with childhood tics and obsessive-compulsive disorder: Transcriptomic data show common enriched innate immune pathways. *Brain Behav. Immun.* 94 308–317. 10.1016/j.bbi.2020.12.035 33422639

[B26] Jones K. Croen L. Yoshida C. Heuer L. Hansen R. Zerbo O. (2017). Autism with intellectual disability is associated with increased levels of maternal cytokines and chemokines during gestation. *Mol. Psychiatry* 22 273–279. 10.1038/mp.2016.77 27217154 PMC5122473

[B27] Kanehisa M. (2019). Toward understanding the origin and evolution of cellular organisms. *Protein Sci.* 28 1947–1951. 10.1002/pro.3715 31441146 PMC6798127

[B28] Kanehisa M. Goto S. (2000). KEGG: Kyoto encyclopedia of genes and genomes. *Nucleic Acids Res.* 28 27–30. 10.1093/nar/28.1.27 10592173 PMC102409

[B29] Kanehisa M. Furumichi M. Sato Y. Ishiguro-Watanabe M. Tanabe M. (2020). KEGG: Integrating viruses and cellular organisms. *Nucleic Acids Res.* 49, D545–D551. 10.1093/nar/gkaa970 33125081 PMC7779016

[B30] Leckman J. Katsovich L. Kawikova I. Lin H. Zhang H. Kronig H. (2005). Increased serum levels of interleukin-12 and tumor necrosis factor-alpha in Tourette’s syndrome. *Biol. Psychiatry* 57 667–673. 10.1016/j.biopsych.2004.12.004 15780855

[B31] Lennington J. Coppola G. Kataoka-Sasaki Y. Fernandez T. Palejev D. Li Y. (2016). Transcriptome analysis of the human striatum in Tourette syndrome. *Biol. Psychiatry* 79 372–382. 10.1016/j.biopsych.2014.07.018 25199956 PMC4305353

[B32] Li M. Santpere G. Imamura Kawasawa Y. Evgrafov O. Gulden F. Pochareddy S. (2018). Integrative functional genomic analysis of human brain development and neuropsychiatric risks. *Science* 362:eaat7615. 10.1126/science.aat7615 PMC6413317 30545854

[B33] Li X. Chauhan A. Sheikh A. Patil S. Chauhan V. Li X. (2009). Elevated immune response in the brain of autistic patients. *J. Neuroimmunol.* 207 111–116. 10.1016/j.jneuroim.2008.12.002 19157572 PMC2770268

[B34] Markiewski M. Lambris J. (2007). The role of complement in inflammatory diseases from behind the scenes into the spotlight. *Am. J. Pathol.* 171 715–727. 10.2353/ajpath.2007.070166 17640961 PMC1959484

[B35] Mataix-Cols D. Isomura K. Perez-Vigil A. Chang Z. Ruck C. Larsson K. (2015). Familial risks of Tourette syndrome and chronic tic disorders. A population-based cohort study. *JAMA Psychiatry* 72 787–793. 10.1001/jamapsychiatry.2015.0627 26083307

[B36] Mittal S. (2020). Tics and Tourette’s syndrome. *Drugs Context* 9 2019–2012. 10.7573/dic.2019-12-2 32273897 PMC7111125

[B37] Morer A. Chae W. Henegariu O. Bothwell A. Leckman J. Kawikova I. (2010). Elevated expression of MCP-1. IL-2 and PTPR-N in basal ganglia of Tourette syndrome cases. *Brain Behav. Immun.* 24 1069–1073. 10.1016/j.bbi.2010.02.007 20193755

[B38] Müller N. (2019). The role of intercellular adhesion molecule-1 in the pathogenesis of psychiatric disorders. *Front. Pharmacol.* 10:1251. 10.3389/fphar.2019.01251 31824303 PMC6883971

[B39] Paschou P. Yu D. Gerber G. Evans P. Tsetsos F. Davis L. (2014). Genetic association signal near NTN4 in Tourette syndrome. *Ann. Neurol.* 76 310–315. 10.1002/ana.24215 25042818 PMC4140987

[B40] Patel S. Dale R. Rose D. Heath B. Nordahl C. Rogers S. (2020). Maternal immune conditions are increased in males with autism spectrum disorders and are associated with behavioural and emotional but not cognitive co-morbidity. *Transl. Psychiatry* 10:286. 10.1038/s41398-020-00976-2 32796821 PMC7429839

[B41] Pringsheim T. Hammer T. (2013). Social behavior and comorbidity in children with tics. *Pediatr. Neurol.* 49 406–410. 10.1016/j.pediatrneurol.2013.08.005 24095577

[B42] Robinson M. McCarthy D. Smyth G. (2010). edgeR: A Bioconductor package for differential expression analysis of digital gene expression data. *Bioinformatics* 26 139–140. 10.1093/bioinformatics/btp616 19910308 PMC2796818

[B43] Sandin S. Lichtenstein P. Kuja-Halkola R. Larsson H. Hultman C. Reichenberg A. (2014). The familial risk of autism. *JAMA* 311 1770–1777. 10.1001/jama.2014.4144 24794370 PMC4381277

[B44] Schafer D. Lehrman E. Kautzman A. Koyama R. Mardinly A. Yamasaki R. (2012). Microglia sculpt postnatal neural circuits in an activity and complement-dependent manner. *Neuron* 74 691–705. 10.1016/j.neuron.2012.03.026 22632727 PMC3528177

[B45] Scharf J. Yu D. Mathews C. Neale B. Stewart S. Fagerness J. (2013). Genome-wide association study of Tourette’s syndrome. *Mol. Psychiatry* 18 721–728. 10.1038/mp.2012.69 22889924 PMC3605224

[B46] Shannon P. Markiel A. Ozier O. Baliga N. Wang J. Ramage D. (2003). Cytoscape: A software environment for integrated models of biomolecular interaction networks. *Genome Res.* 13 2498–2504. 10.1101/gr.1239303 14597658 PMC403769

[B47] The Gene Ontology Consortium. (2019). The gene ontology resource: 20 years and still GOing strong. *Nucleic Acids Res.* 47 D330–D338. 10.1093/nar/gky1055 30395331 PMC6323945

[B48] Tick B. Bolton P. Happe F. Rutter M. Rijsdijk F. (2016). Heritability of autism spectrum disorders: A meta-analysis of twin studies. *J. Child Psychol. Psychiatry* 57 585–595. 10.1111/jcpp.12499 26709141 PMC4996332

[B49] Vargas D. Nascimbene C. Krishnan C. Zimmerman A. Pardo C. (2005). Neuroglial activation and neuroinflammation in the brain of patients with autism. *Ann. Neurol.* 57 67–81. 10.1002/ana.20315 15546155

[B50] Voineagu I. Wang X. Johnston P. Lowe J. Tian Y. Horvath S. (2011). Transcriptomic analysis of autistic brain reveals convergent molecular pathology. *Nature* 474 380–384. 10.1038/nature10110 21614001 PMC3607626

[B51] Weber-Stadlbauer U. (2017). Epigenetic and transgenerational mechanisms in infection-mediated neurodevelopmental disorders. *Transl. Psychiatry* 7:e1113. 10.1038/tp.2017.78 28463237 PMC5534947

[B52] Wickham H. (2016). *ggplot2: Elegant grapics for data analysis.* New York, NY: Springer-Verlag. 10.1007/978-3-319-24277-4

[B53] Wile D. Pringsheim T. (2013). Behavior therapy for Tourette syndrome: A systematic review and meta-analysis. *Curr. Treat Options Neurol.* 15 385–395. 10.1007/s11940-013-0238-5 23645295

[B54] Wright C. Shin J. Rajpurohit A. Deep-Soboslay A. Collado-Torres L. Brandon N. (2017). Altered expression of histamine signaling genes in autism spectrum disorder. *Transl. Psychiatry* 7:e1126. 10.1038/tp.2017.87 28485729 PMC5534955

[B55] Yu G. Wang L. Han Y. He Q. (2012). clusterProfiler: An R package for comparing biological themes among gene clusters. *OMICS* 16 284–287. 10.1089/omi.2011.0118 22455463 PMC3339379

