# Supplementary figures and images for: Common targetable inflammatory pathways in brain transcriptome of autism spectrum disorders and Tourette syndrome
Source: Front Neurosci. 2022 Dec 15;16:999346. doi: 10.3389/fnins.2022.999346 (PMC9799059; doi:10.3389/fnins.2022.999346)

# ASD - Hierarchical clustering analyses

**A**

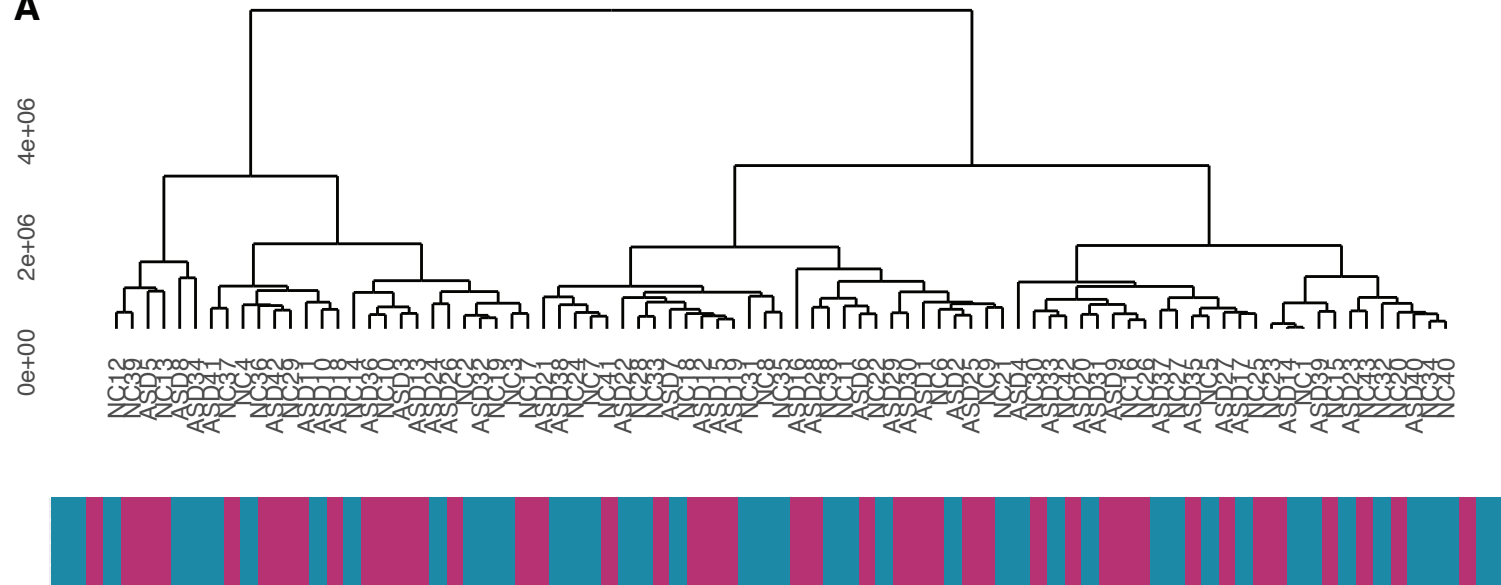

**B**

**Variance Histogram**

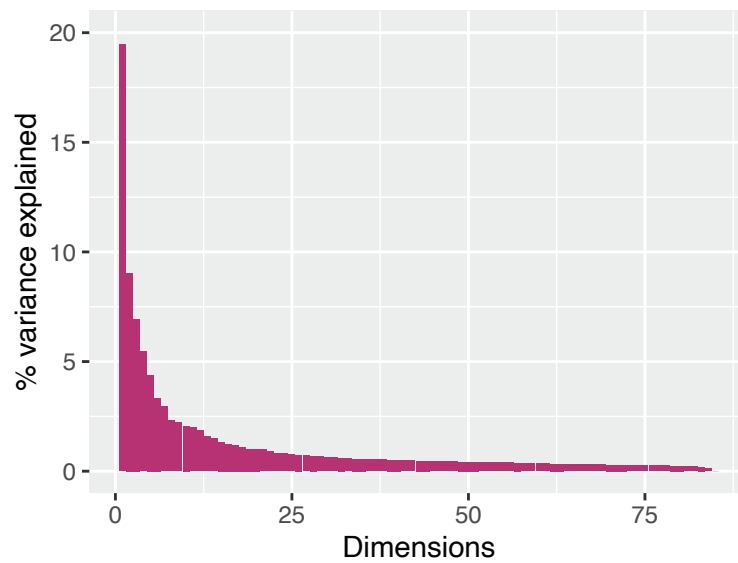

**C**

**Principal Component Analysis**

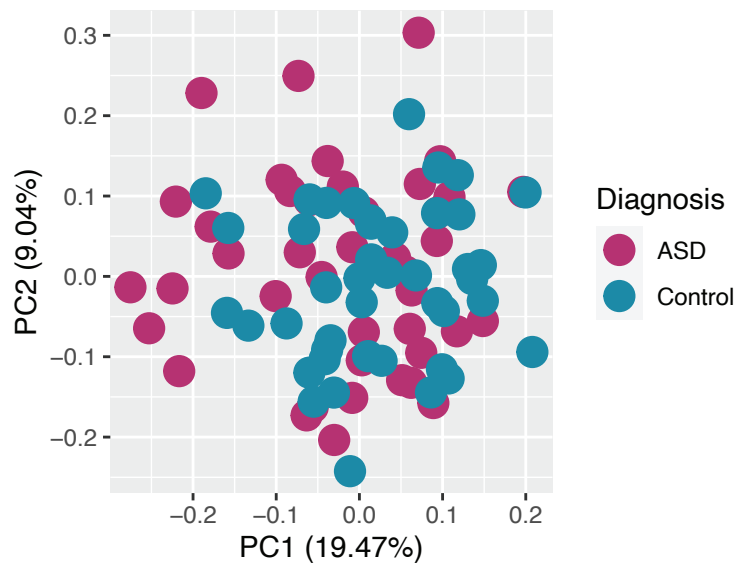

Supplement: Supplementary Figure 1 — Transcriptional clustering of ASD patients and normal controls. Cluster analyses identifying transcriptional differences within the autism-spectrum disorder (ASD; pink) patients and normal controls (NC; blue) using Euclidean distances. (A) Hierarchal cluster dendrogram using the agglomerative method. (B) Variance histogram identifies the amount of variance (y-axis) explained by each principal component (x-axis; dimension). (C) Principal component analysis (PCA) scatter plot of PC2 (y-axis) and PC1 (x-axis) which account for most of the variance in the data set. ASD, autism-spectrum disorder (n = 42), NC, normal control (n = 43). [file Image_1.PDF]

# TS - Hierarchal clustering analyses

**A**

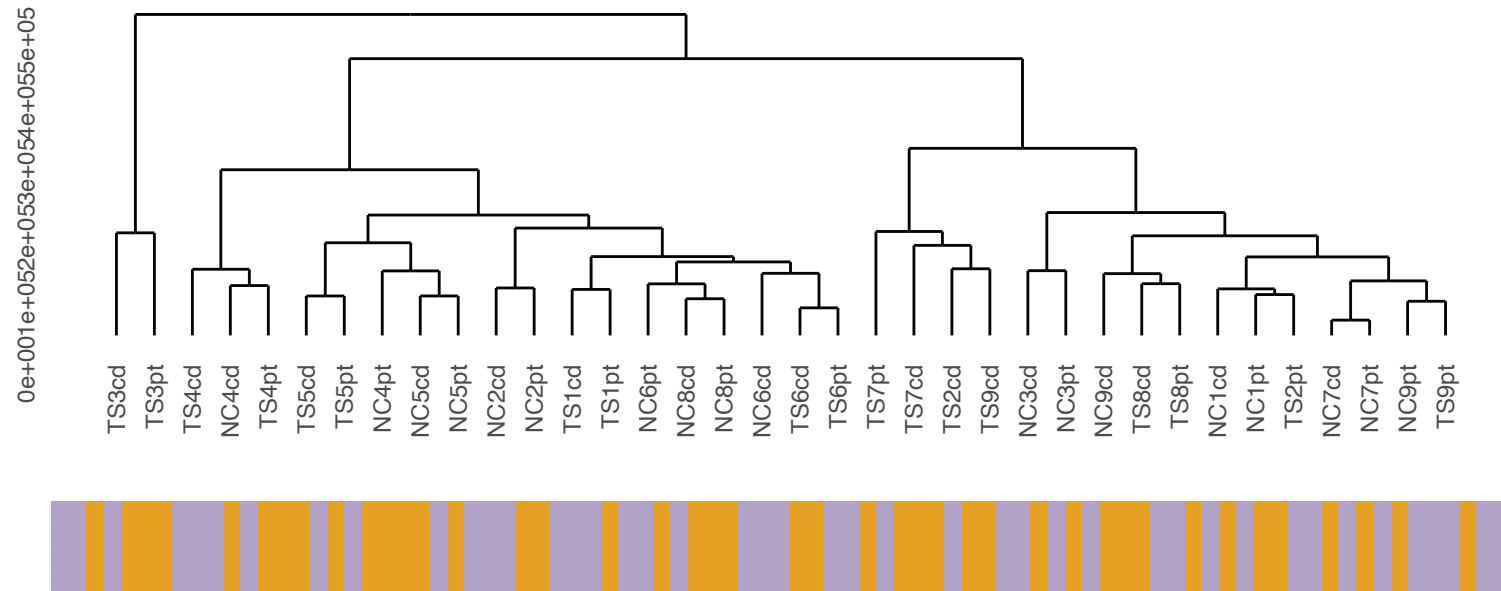

**B**

**Variance Histogram**

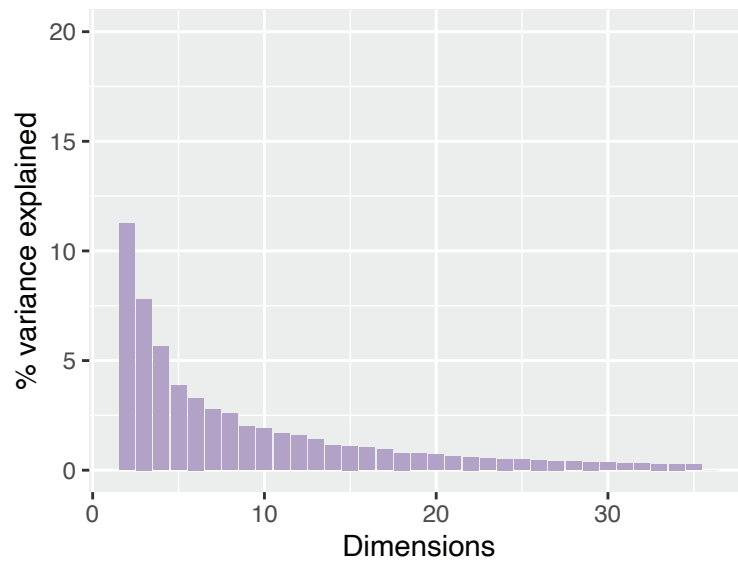

**C**

**Principal Component Analysis**

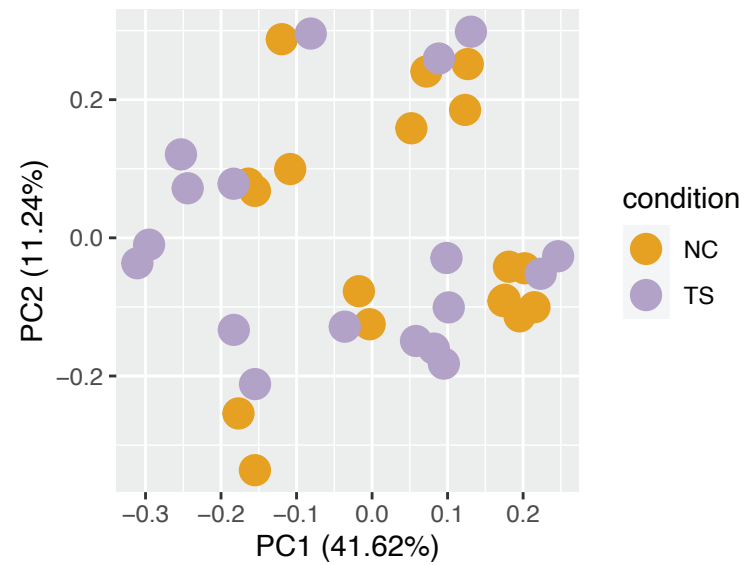

Supplement: Supplementary Figure 2 — Transcriptional clustering of TS and normal controls. Cluster analyses identifying transcriptional differences within individuals with Tourette syndrome (TS; purple) and normal controls (NC; yellow) using Euclidean distances. (A) Hierarchal cluster dendrogram using the agglomerative method. (B) Variance histogram identifies the amount of variance (y-axis) explained by each principal component (x-axis; dimension). (C) Principal component analysis (PCA) scatter plot of PC2 (y-axis) and PC1 (x-axis) which account for most of the variance in the data set. TS, Tourette syndrome (n = 9), NC, normal control (n = 9). [file Image_2.PDF]

# ASD – GO terms (FDR <0.05 pathways)

DGE parameters: FDR < 0.05, 81 pathways

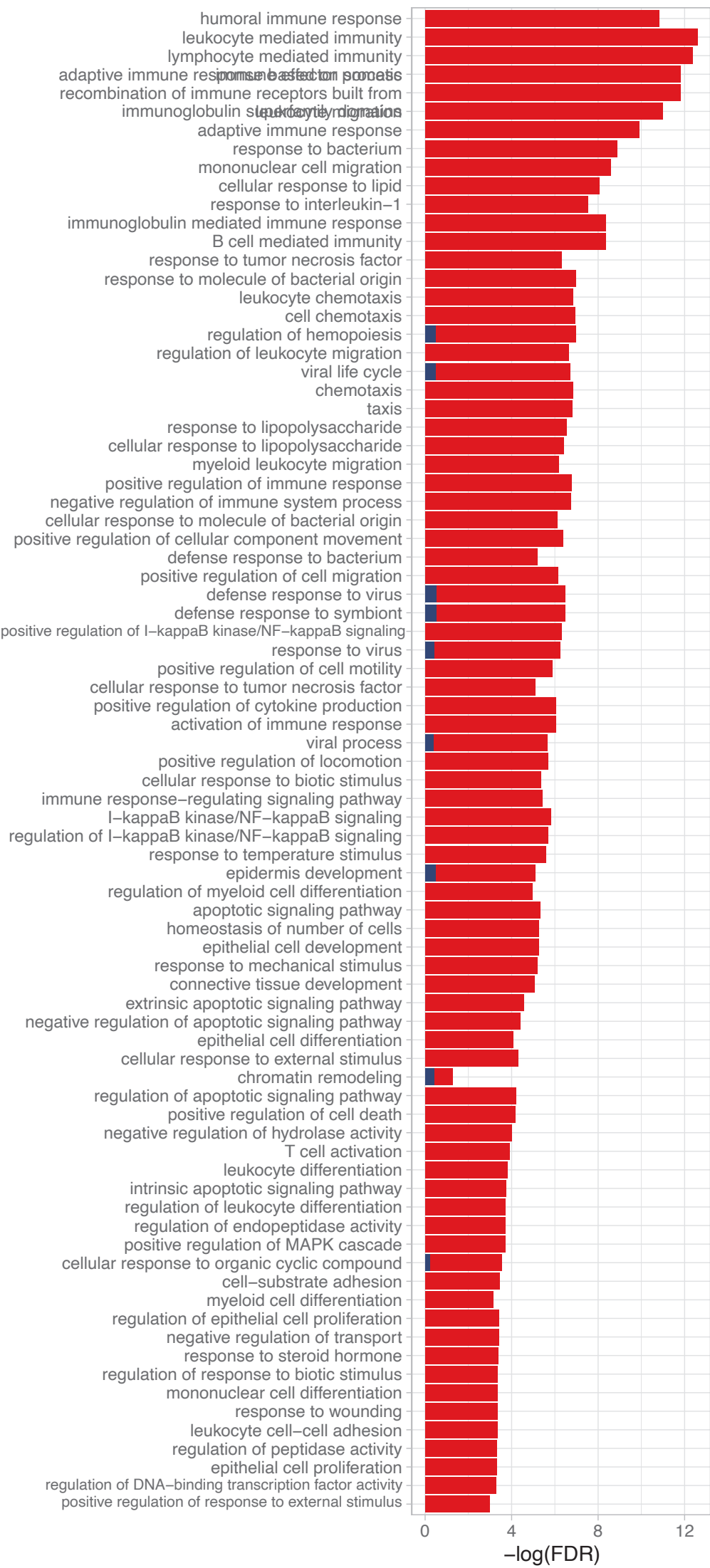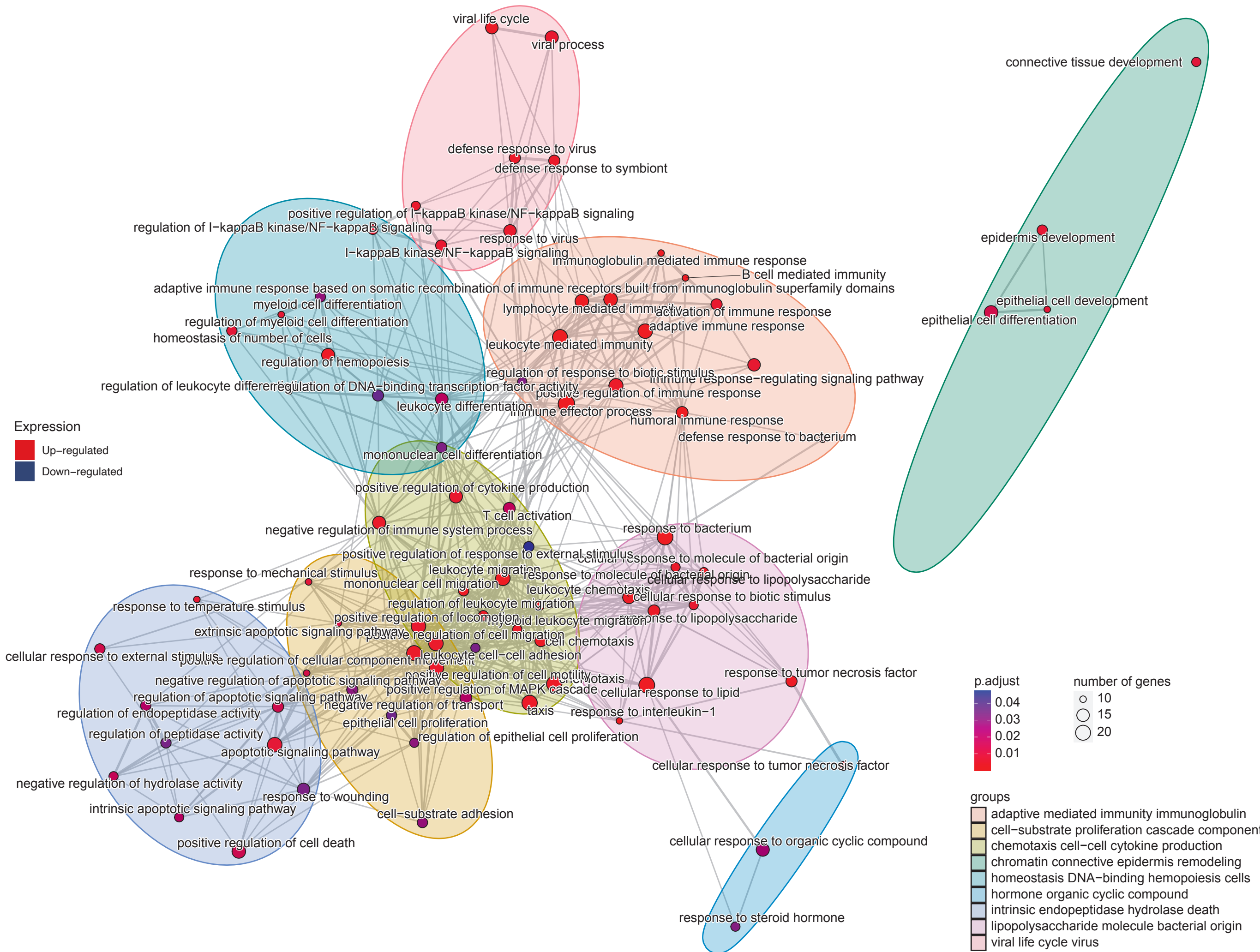

Supplement: Supplementary Figure 3 — Autism spectrum disorders (ASD) Gene Ontology (GO) enrichment analysis. GO enrichment analysis (FDR/p.adjust < 0.05) of the top differentially expressed genes (P value < 0.05) in ASD. Statistical significance of the pathway (FDR) enriched is shown on the y-axis, while the enriched term is shown on the x-axis. [file Image_3.PDF]

# TS – GO terms (FDR <0.05 terms)

Pathways

Expression

Up-regulated

Down-regulated

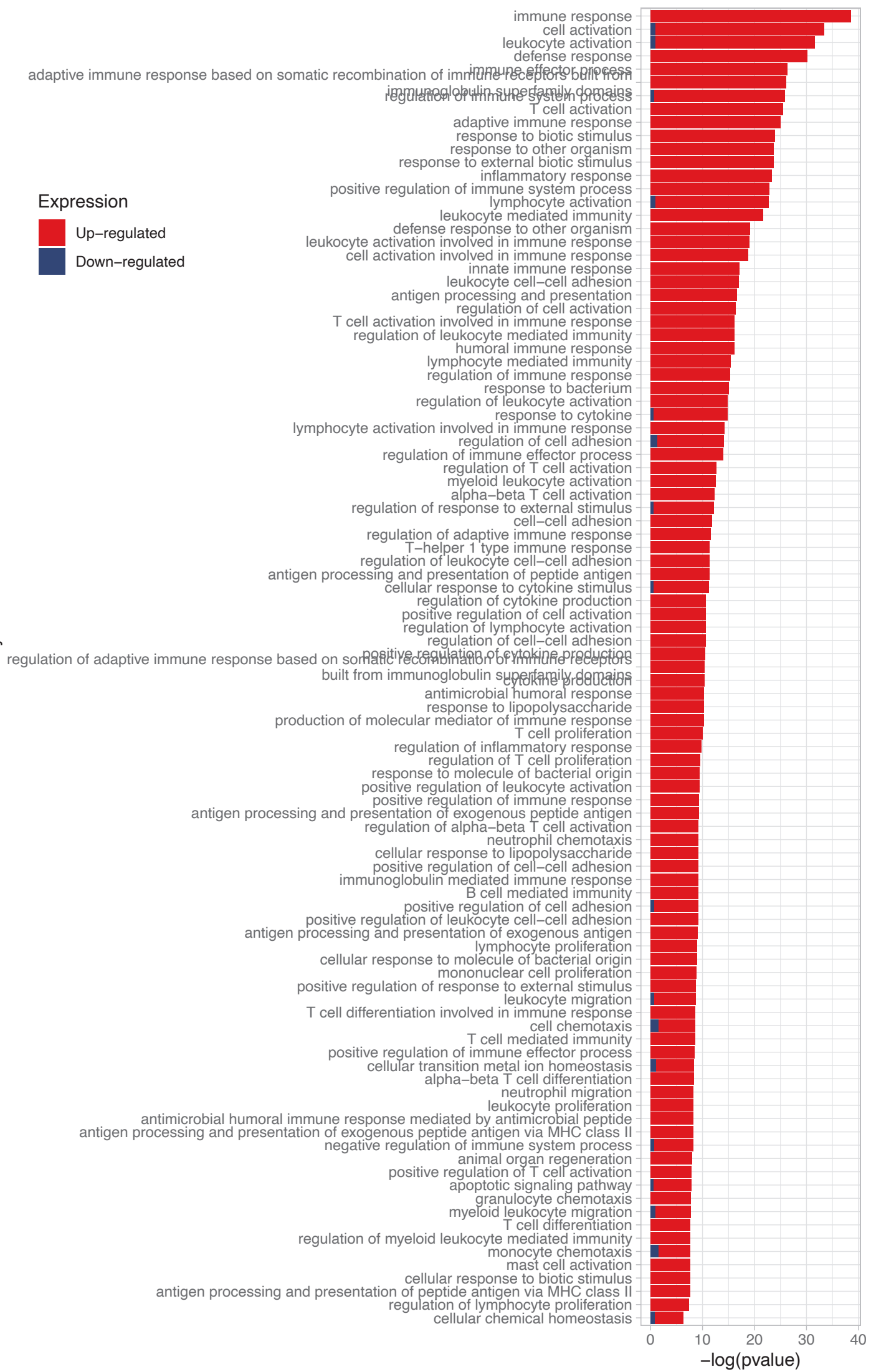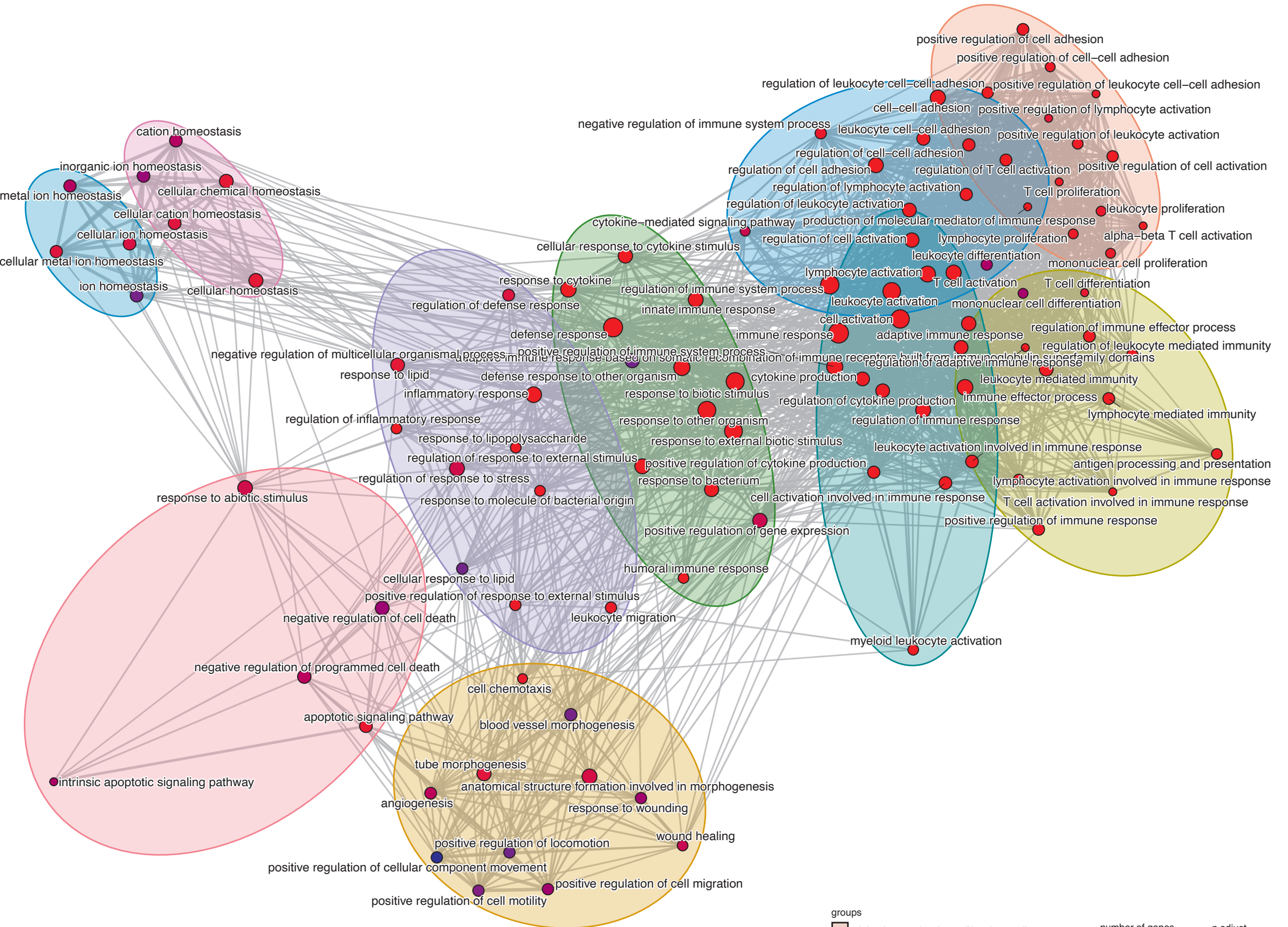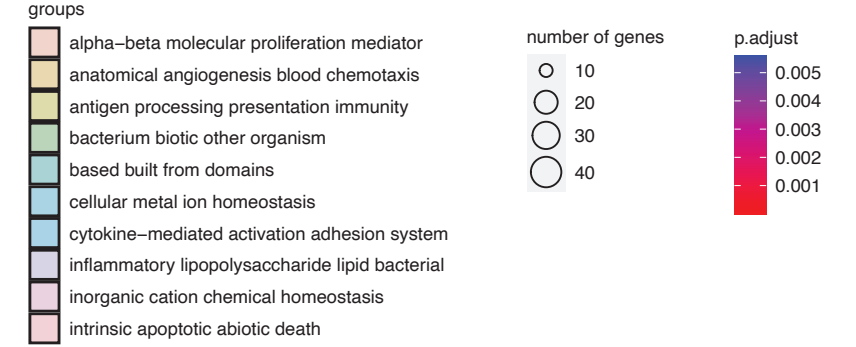

Supplement: Supplementary Figure 5 — Tourette syndrome Gene Ontology (GO) enrichment analysis. GO enrichment analysis (FDR/p.adjust < 0.05) of the top differentially expressed genes (P value < 0.05) in Tourette syndrome. Statistical significance of the pathway (FDR) enriched is shown on the y-axis, while the enriched term is shown on the x-axis. [file Image_5.PDF]
