# Supplementary material for: Common targetable inflammatory pathways in brain transcriptome of autism spectrum disorders and Tourette syndrome
Source: Front Neurosci. 2022 Dec 15;16:999346. doi: 10.3389/fnins.2022.999346 (PMC9799059; doi:10.3389/fnins.2022.999346)

ASD – KEGG pathways (FDR <0.05 terms)

DGE parameters: FDR < 0.05, 9 pathways total

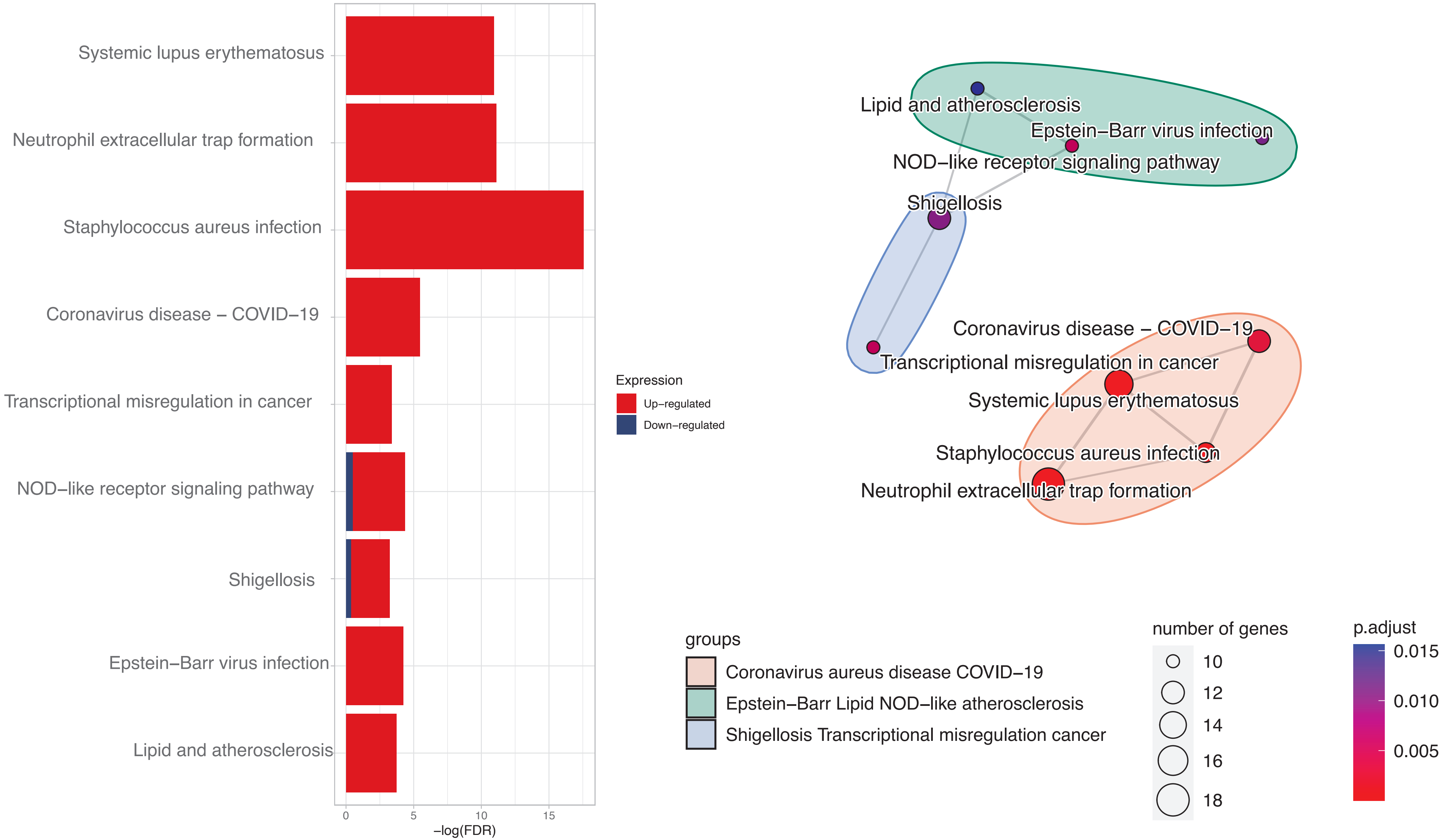

Supplement: Supplementary Figure 4 — Autism spectrum disorders (ASD) Kyoto Encyclopedia of Genes Genomes (KEGG) enrichment analysis. KEGG enrichment analysis (FDR/p.adjust < 0.05) of the top differentially expressed genes (P value < 0.05) in ASD. Statistical significance of the pathway (FDR) enriched is shown on the y-axis, while the enriched pathway is shown on the x-axis. [file Image_4.PDF]
