# Supplementary material for: Common targetable inflammatory pathways in brain transcriptome of autism spectrum disorders and Tourette syndrome
Source: Front Neurosci. 2022 Dec 15;16:999346. doi: 10.3389/fnins.2022.999346 (PMC9799059; doi:10.3389/fnins.2022.999346)
Supplement: Supplementary file 6 [file Table_1.DOCX]

**Supplementary Table 1: Demographic and clinical variables**

| Variables | Patients | Normal controls |
| --- | --- | --- |
| **Autism spectrum disorders** | (n=42) | (n=43) |
| Age |  |  |
| Mean (SD) | 26.38 (17.46) | 28.62 (17.05) |
| Minimum-maximum | 2-67 | 4-60 |
| Sex |  |  |
| Male | 33 (78.57%) | 34 (79.07%) |
| Female | 9 (21.43%) | 9 (20.93%) |
|  |  |  |
| **Tourette syndrome** | (n=9) | (n=9) |
| Age |  |  |
| Mean (SD) | 62.77 (16.73) | 58 (15.77) |
| Minimum-maximum | 41-86 | 28-82 |
| Sex |  |  |
| Male | 5(55.55%) | 5(55.55%) |
| Female | 4(44.44%) | 4(44.44%) |
